# 

**Published:** 1893-01

**Authors:** 


IN WRITING TO THE JOURNAL'S ADVERTISERS PLEASE MENTION WHERE YOU SAW THE ADVERTISEMENT.

LIBRARY Indexed S. G. O.

ESTABLISHED 1855. SUBSCRIPTION, $2.00.

### ATLANTA MEDICAL AND SURGICAL JOURNAL.

OLD SERIES, VOL. XXVI. NEW SERIES, VOL. IX. } ATLANTA, GA., JANUARY, 1893. No. 11.

LUTHER B. GRANDY, M. D., MANAGING EDITOR.

M. B. HUTCHINS, M. D. BUSINESS MANAGER.

